# Assessing Influence Mechanism of Green Utilization of Agricultural Wastes in Five Provinces of China through Farmers’ Motivation-Cognition-Behavior

**DOI:** 10.3390/ijerph17103381

**Published:** 2020-05-12

**Authors:** Liying Yu, Hongda Liu, Ardjouman Diabate, Yuyao Qian, Hagan Sibiri, Bing Yan

**Affiliations:** 1 School of Management, Shanghai University, Shanghai 200444, China; yuliying@shu.edu.cn (L.Y.); liuhoda@163.com (H.L.); diabateardjouman@yahoo.fr (A.D.); 2 School of Foreign Languages, Shanghai University, Shanghai 200444, China; qyy19720498@shu.edu.cn; 3 School of International Relations and Public Affairs, Fudan University, Shanghai 200433, China; shagan17@fudan.edu.cn; 4 School of Urban Railway Transportation, Shanghai University of Engineering Science, Shanghai 201620, China

**Keywords:** agricultural waste, green utilization, influence mechanism, farmers, motivation-cognition-behavior, structural equation model

## Abstract

Using the theory of motivation, and the theory of planned behavior, this study establishes the “motivation-cognition-behavior” model of green utilization of agricultural waste from the perspective of farmers. In the motivational dimension, eight motivational factors were determined in three sub-dimensions of extrinsic motivation. In the cognitive dimension, three sub-dimensions of subjective norms, behavioral attitude, and perceived behavioral control are also determined. In the behavioral dimension, two sub-dimensions of utilization intention and utilization behavior are specified. Methodologically, a questionnaire on the green utilization of agricultural waste of 704 peasant households in five provinces of Jiangsu, Anhui, Shaanxi, Gansu, and Sichuan was administered. With the help of the structural equation model, the influence path and the internal mechanism was then analyzed. It is shown that: (1) in relation to the “motivational dimension → cognitive dimension,” extrinsic motivation significantly promotes the cultivation of farmers’ subjective norms, in which positive broken windows theory has a positive effect. In contrast, negative broken windows theory has a negative one. In intrinsic motivation, the behavior attitude of farmers is negative. In the response analysis, farmers can realize that their ability, self-efficacy, response efficacy, and response cost all have a positive impact on farmers’ perceived behavioral control. (2) In relation of the “cognitive dimension → behavioral dimension,” behavioral attitude slightly hinders utilization intention, while subjective norms and perceived behavioral control all contribute to a stronger utilization intention; the utilization intention maintains a positive correlation with the utilization behavior.

## 1. Introduction

Agricultural waste is a general term for waste that includes agricultural production, agricultural product processing, animal husbandry, and pollutant discharge of farmers. From the perspective of energy conversion, it is the share of energy loss from the production of agricultural resources. For years, China has been committed to promoting the utilization of agricultural waste and has since achieved some remarkable results, taking the typical waste—straw, as an example—nad transforming it into feed as the main utilization mode, and into fodder and energy [1]. However, there are still many problems in the process of the green utilization of agricultural waste, such as the weak foundation of green utilization, farmers’ poor awareness, and high input costs, among others. Farmers are the mainstay of the green utilization of agricultural waste. Therefore, if there is a cognitive bias in the utilization of waste, we can only achieve short-term results by merely relying on the policy to drive and supervise the comprehensive utilization of agricultural waste. That is to say, to reverse the decline in the green utilization of agricultural waste from the root cause, farmers must play a key role in reconstructing the environment for the utilization of agricultural waste, and shifting the utilization of waste from being driven by external policies to being driven by farmers’ internal recognition. Thus, in terms of promoting the green utilization of agricultural waste, more research is required to clarify the key influencing factors of green utilization of agricultural waste and to explore the psychological mechanisms of farmers’ decision-making.

Researchers have always valued the issue of resource utilization of agricultural waste. Marousek [2] analyzed the utilization of agricultural waste in Europe, emphasizing that under unequal commercial conditions, farmers rely entirely on government subsidies to generate interest. Once the incentives wane and policies are withdrawn, farmers will incinerate the waste without considering the ecological consequences. Stana and Medek [3] explored prospects for the energy utilization of agricultural waste and believed that optimizing the incineration process could improve utilization efficiency. Syamsu and Karim [4] studied the policy-design path for transforming agricultural waste into feed, emphasizing the necessity of group feeds and the restructuring of an agricultural waste disposal system. In general, many countries attach great importance to the damage caused by the negative disposal of agricultural waste [5,6], and the level of resource utilization of agricultural waste is relatively high [7,8]. Nevertheless, the utilization of agricultural waste is inevitably supported by substantial government investment, and the enthusiasm of farmers themselves is not strong. The reason for this is that agricultural waste belongs to the farmers, while any related governance has the attributes of public goods. The latter has significant non-competitive and non-exclusive characteristics. Hence, the disposal activities of the waste are still dominated by the government when the market mechanism of waste has not been sound enough [9]. Although the government can effectively guide the farmers’ behavior to a certain extent, in reality, the utilization intention will sometimes be against practical action [10].

Chinese scholars study the green utilization of agricultural waste mainly from the perspective of government, farmers, and enterprises. Gao [11] believes that the current administrative regulations on agricultural waste are too monotonous, that the target is misplaced, and that their interests are one-sided. These problems not only raise the cost of governance but also make it hard to meet people’s expectations. Yan [12] analyzed the welfare response to farmers’ resource utilization of straw and pointed out the importance of the popularization of preferential policies and the awareness of environmental regulations on the resource utilization of agricultural waste. Based on biological utilization promotion policy [13] and policy mix [14], Renjiqin and Tong Hongzhi studied farmers’ adoption intention of protective tillage behaviors. They believed that the government should subsidize farming costs, intervene in the utilization of agricultural waste by leverage mechanism, and encourage farmers to adopt protective tillage technology to reduce the damage by agricultural waste. Jiang [15] stressed that farmers need strong incentive policies to use agricultural biomass waste, and the increase in farmers’ willingness to participate stems from the improvement of their interests. Zhang [16] found that in the process of agricultural waste recycling, farmers’ behavior will be affected by the relationship between cadres and masses in the village. Chen [17] studied the behavior of enterprises in the process of agricultural waste utilization, emphasizing that effective fund-raising is conducive to increase the willingness of enterprises to participate in the improvement of farmers’ behavior. From the perspective of China’s practice, farmers’ participation motivation is insufficient [18], and is easily influenced by the external environment and other farmers [19], thus making it difficult to rely solely on the government to promote the green utilization of agricultural waste [20]. This paper, thus, takes farmers as the research object to probe the mechanism of farmers’ participation in agricultural waste utilization systems, and is set out to answer the following research questions: how to give full play to farmers’ subjective initiative, in order to awaken the intrinsic motivation of farmers’ green utilization behavior? How to construct an external policy environment, thus forming a positive external driving force? The authors hope that the research results can turn the passive green utilization of agricultural waste into the active one. In the case of farmers’ green utilization of agricultural waste, this paper introduces the motivation theory and the theory of planned behavior. It analyzes the farmers’ motivation for the green utilization of agricultural waste from the perspective of farmers’ cognition-behavior. Considering the double broken window effect, the paper then analyzes the factors influencing the green utilization of agricultural waste, thus using a structural equation model to determine key factors and critical paths. Finally, from the perspective of the reconstruction of farmers’ values, the paper proposes countermeasures and suggestions to break the bottleneck of agricultural waste utilization.

## 2. Theoretical Analysis and Mechanism

### 2.1. The “Motivation-Cognition-Behavior” Theoretical Framework for the Green Utilization of Agricultural Waste

The theory of planned behavior was first proposed by American scholars Ajzen and Madden [21]. Based on psychology [22], they constructed a “cognition-behavior” logical model, emphasizing that cognition is the driving force of behavior and also the antecedent factor in triggering behavior intention, and behavior intention further leads to actual behavior decisions. The model is divided into two dimensions: cognition and behavior. The cognitive dimension mainly reflects individual cognition, including three sub-dimensions: subjective norms, behavioral attitude, and perceived behavioral control. Subjective norms reflect the influence of the external environment (including other groups, policies, ethics, and conventions, among others) imposed on the actor’s decision-making. The behavioral attitude demonstrates the actor’s thoughts and attitudes toward the target activities. While perceived behavioral control is the actor’s perception of the difficulty of the target activity, which is reflected by an individual’s specific ability to complete the activity. The behavior dimension consists of two sub-dimensions: intention and practical action. The behavior intention is the ultimate intention and course of action influenced by the subjective norms, behavioral attitude, and perceived behavioral control, which will further influence and guide the actor’s ultimate practical actions. It can be seen that the essence of the theory of planned behavior is that the thought determines the action, and the plan guides the behavior. It conjectures actor’s thoughts through the measurements in the cognitive dimension, thereby displaying the influence of psychological activity on decision-making.

With the help of the theory of planned behavior, researchers demonstrated the factors that influence farmers in agricultural production activities [23,24]. However, the source of farmers’ intention was still neglected in the research. It only relied on the scale to analyze farmers’ performance in the cognitive dimension but failed to dig out the source and driving factors of the cognitive dimension of the theory of planned behavior. Thus, while the research explained the intervention effect of farmers’ psychological activities on decision-making and the implementation methods, it ignored the factors affecting farmers’ actual “psychological activities.” According to the theory of planned behavior, the subjective norms, behavioral attitude, and perceived behavioral control in the cognitive dimension are the results of farmers’ comprehensive evaluation of the external environment, as well as their self-motivation and self-conditions. In essence, subjective norms are determined by extrinsic motivation. While behavioral attitude is decided by intrinsic motivation, the perceived behavioral control shows farmers’ execution status. This paper, therefore, believes that the theory of planned behavior can be applied to the research on farmers’ green utilization of agricultural waste. Nevertheless, since the cognitive process will be influenced by motivation, the motivation of farmers’ green behavior should be explored.

Motivation theory focuses on the reasons that influence people’s behavior [25,26,27]. There is also intrinsic and extrinsic motivation theory [26,28], protection motivation theory [29], Attention-Relevance-Confidence-Satisfaction(ARCS) motivation theory [30], and learning motivation theory [31], among others. Among them, the intrinsic and extrinsic motivation theory can better explain the psychological activities of the behavior subject when they are affected by the internal and external environment [26], and the protective motivation theory can interpret the cognitive and coping process of the behavior subject after obtaining the subjective information [29]. Because the theory of protection motivation embodies the internal thinking process of the behavior subject, Cai [32] combines the theory of internal motivation and puts forward the theory of self-protection motivation. In the green utilization of agricultural waste, farmers will be affected by external environments and internal thinking, and will also carry out a threat assessment and response assessment on the utilization process. Therefore, the behavior of farmers is essentially the result of internal and external motivation and protection motivation. The internal motivation and threat analysis together form farmers’ self-protection motivation.

Farmers’ motivation for the green utilization of agricultural waste includes extrinsic motivation, intrinsic motivation, and protection motivation. Extrinsic motivation [28] means that external conditions exert impact on individuals and encourage them to participate in the green utilization activity. Likewise, the subjective norms in the cognitive dimension reflect the final result or state of the influence of external pressure (motivation) on individual behavior. Therefore, extrinsic motivation theory can explain the manifestation and change of subjective norms, which is an “extrinsic motivation → subjective norm” influence path.

Therefore,

**Hypothesis** **H1.**  External motivation has a positive effect on farmers’ subjective norms.

The intrinsic motivation is embodied in an individual’s preference for things, which means an individual does not choose to conduct an activity or decision by himself/herself without the interference of environmental and external forces. The behavioral attitude in the cognitive dimension is the reflection of farmers’ utilization of agricultural waste, which is the external manifestation of intrinsic motivation. Hence, there is an “intrinsic motivation → behavioral attitude” path in this dimension.

In the measurement of intrinsic motivation, the threat assessment [33] of bad behavior proposed by the protection motivation theory can reveal farmers’ intrinsic psychological activities. The farmers will participate in the agricultural waste disposal considering the consequences of bad behaviors in the disposal process. Therefore, there is an “intrinsic motivation (threat assessment) → behavioral attitude” path in this dimension.

Therefore,

**Hypothesis** **H2.**  Intrinsic motivation (threat assessment) has a positive effect on the behavior attitude of farmers.

The response analysis of the adaptability of target behavior [33] proposed by the protection motivation theory is the evaluation of the subject’s ability. The result of the analysis is the subject’s ability to perceive behavioral control, that is, whether the individual can cope with the challenge of the target activity. Therefore, there is a “response analysis → perceived behavior control” path.

Therefore,

**Hypothesis** **H3.**  Response analysis has a positive effect on the perceived behavior control of farmers.

In the theory of planning behavior [22], it is generally believed that planning will have a positive effect on behavior. The three sub-dimensions of cognitive dimensions (subjective norms, behavioral attitude, and perceived behavioral control) are produced and controlled by multiple motivations. These dimensions ultimately affect the behavior of the subject.

**Hypothesis** **H4.**  *Cognition has a positive effect on behavior.*

Combined with the above analyses, this paper forms a “motivation-cognition-behavior” theoretical structure. From the perspective of extrinsic motivation, intrinsic motivation, and response motivation, we can better clarify the mechanisms behind how farmers’ psychological decision-making influences their green utilization of agricultural waste.

### 2.2. Analysis of the Broken Window Effect on the Extrinsic Motivation of Farmers’ Green Utilization Behavior

In the process of farmers’ processing agricultural waste, the external environment is mainly composed of the government and other farmers. However, due to the stronger group attributes of farmers, the influence of environmental interventions such as government regulations or incentives are significantly weaker than that of the influence mechanism among farmers. Farmers have a lower level of education and weaker information acquisition capabilities; hence, their understanding of policy orientation largely stems from the attitudes of information carriers. Most of this information is transmitted in the farmers’ group, so other farmers mainly stimulate farmers’ extrinsic motivation for the green utilization of agricultural waste. This has caused a notable broken window effect in the influence mechanisms existing within the farmer group.

The broken windows theory was first proposed by Wilson [34]. The theory posits that if one overlooks a bad phenomenon, it will break the constraints of rules and induce people to follow the crowd. If farmers are allowed to adopt agricultural waste disposal that will cause damage to the environment, then a bad transmission mechanism will be formed, leading more farmers to participate in negative disposal activities. This is defined as a negative broken window effect in this paper. In the green utilization of agricultural waste, a positive broken window effect also exists: first, as agricultural waste is an idle resource, the disposal of it is beneficial to the environment on the one hand, and brings about the new economy (such as green agriculture) on the other; second, the rational thinking of “propensity to the benefits” catalyzes more farmers to participate in agricultural waste utilization activities. Farmers who participated in the green utilization of agricultural waste in the early stage and profited will have caused an opinion–leader effect, which triggers more farmers to form a positive idea about “making money without trouble,” thus helping to promote green utilization. Therefore, the broken window effect also plays a positive role in farmers’ decision-making, that is, the positive broken window effect. The positive and negative broken window effects among farmers together constitute the extrinsic motivation for farmers to participate in the green utilization of agricultural waste.

The positive and negative broken window effects are essentially a kind of extrinsic motivation, which means that farmers are affected by external farmer groups and thus form positive motivations or negative thoughts. This process can be regarded as extrinsic motivations affecting farmer’s intentions.

### 2.3. An Analysis Framework Based on the “Motivation-Cognition-Behavior” Model

Based on the above analysis, this paper proposes the “motivation-cognition-behavior” analysis framework for green utilization of agricultural waste. In the process of green utilization of agricultural waste, intrinsic and extrinsic motivation, threat analysis, and response analysis jointly reflect farmers’ participation motivation. Subjective norms, behavioral attitudes, and perceived behavioral control comprehensively demonstrate farmers’ cognition, while utilization intention and utilization behavior display farmers’ actual behavior.

There are 13 related variables in farmers’ “motivation-cognition-behavior” in the green utilization of agricultural waste, including eight motivational factors, three cognitive sub-dimensional variables, and two behavioral sub-dimensional variables.

**Figure 1:**
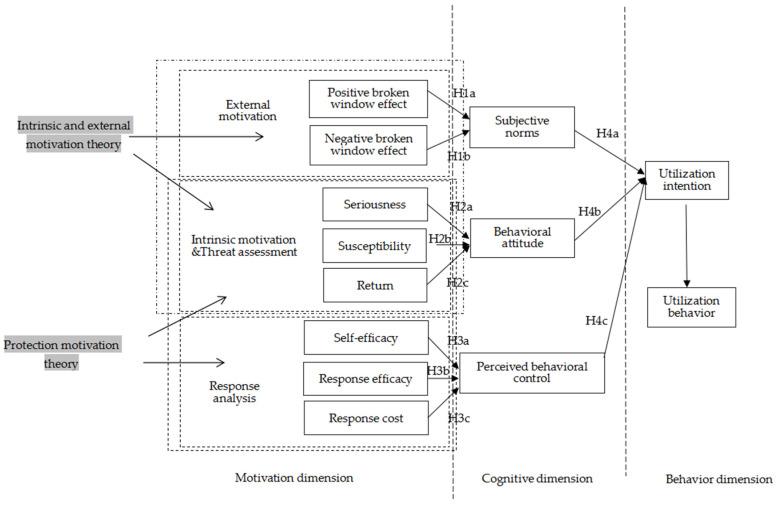
Analysis Framework.

(1) Extrinsic motivation in the motivational dimension. Considering the double broken window effect, two extrinsic motivational factors are defined: positive broken window and negative broken window variables. The positive broken window effect is characterized by farmers’ perception of the external environment in which agricultural waste are used, specifically: the extrinsic motivation formed by farmers when other people obtain considerable utilization benefits; whether farmers’ incineration activities will dwindle under a good environmental governance and supervision system; and when actively participating in green utilization, whether farmers will have an impact on the outside world, that is, the spread of positive broken window effects. Negative broken window effect is characterized by farmers’ perception of the external environment of agricultural waste incineration, specifically, to what degree will farmers be influenced by bad behaviors and spread their bad behaviors when the governance rules are broken, or under bad environmental governance.
  Therefore, the sub hypothesis is as follows:
  **H1a.**  Positive broken window has a positive effect on subjective norms;
  **H1b.**  Negative broken window has a negative effect on subjective norms.
(2) Intrinsic motivation in the motivational dimension. The threat analysis in the protection motivation theory means that farmers consider comprehensively the adverse effects and benefits of the negative disposal of agricultural waste, which is essentially a quantitative manifestation of farmers’ intrinsic motivation [35]. As a result, the threat assessment of protection motivation and intrinsic motivation is the same. The measurements of threat assessment: threat assessment = (seriousness + susceptibility)-return [33], in which the seriousness means the consequences (causing damage) resulted from farmers’ negative disposal of agricultural waste, the susceptibility equals to the possibility of vicious consequences caused by negative disposal of agricultural waste, and the return is the benefit from the negative disposal of agricultural waste. Therefore, three intrinsic motivation factors are defined in the measurements of intrinsic motivation: seriousness, susceptibility, and return.
  Therefore, the sub hypothesis is as follows:
  **H2a.**  Seriousness has a positive effect on behavior attitude;
  **H2b.**  Susceptibility has a positive effect on behavior attitude;
  **H2c.**  Return has a negative effect on behavior attitude.
(3) Response analysis in the motivational dimension. According to the measurements of response analysis in the protection motivation theory: response analysis = (self-efficacy + response efficacy)-response cost [33]. Self-efficacy is farmers’ judgment on the conditions of green utilization of agricultural waste, and they can clarify their actual ability and participation status; the response efficiency is characterized by the benefits given to farmers by the green utilization of agricultural waste; the response cost is the farmers’ understanding of the cost of green utilization of agricultural waste. Therefore, three response motivational factors are defined in the measurements of response analysis: self-efficacy, response efficacy, and reflection cost.
  Therefore, the sub hypothesis:
  **H3a.**  Self-efficacy has a positive effect on perceived behavior control;
  **H3b.**  Response efficacy has a positive effect on perceived behavior control;
  **H3c.**  Response cost has a negative effect on perceived behavior control.
(4) Cognitive dimension. According to the theory of planned behavior, three cognitive sub-dimensional variables are defined in this dimension: subjective norms, behavioral attitudes, and perceived behavioral control. The subjective norms mean how deeply farmers are affected by the external environment when participating in decision-making. Behavioral attitude stands for the farmers’ own efforts to participate in activities. Moreover, perceived behavioral control denotes how fully farmers control their capabilities.
  Therefore, the sub hypothesis:
  **H4a.**  Subjective norms have a positive effect on utilization intention;
  **H4b.**  Behavior attitude has a positive effect on utilization intention;
  **H4c.**  Perceived behavior control has a positive effect on utilization intention.
(5) Behavioral dimension. According to the theory of planned behavior, two behavioral sub-dimensional variables are defined in this dimension: utilization intention and utilization behavior, which indicate farmers’ willingness to participate in straw utilization and their actual participation, respectively. See Figure 1 for the above analysis:

## 3. Data Source and Descriptive Analysis

### 3.1. Data Source

Although there are many types of agricultural waste, straw is a typical agricultural waste in rural China [36]. The study was conducted in five provinces in China, namely: Jiangsu, Anhui, Shaanxi, Gansu, and Sichuan.

#### 3.1.1. Overview of the Study Area with Respect to Agricultural Activities

Three main reasons influenced the choice of the five provinces as the survey areas. Firstly, the five provinces are the central provinces of China’s grain regions. To be precise, Jiangsu and Anhui represent the plain areas of the middle and lower reaches of the Yangtze River. Shaanxi and Gansu are the main grain-producing areas of the Loess Plateau, the Huang-Huai Region, and the northwest region, and Sichuan is the major grain-producing province of the southwest region. The five provinces have a complete range of crops, basically covering the main crop types in China, and the planting terrain (plain, middle plateau, and highland, etc.) also basically represents the topography of China’s main planting areas. Thus, we can reflect the reality of China’s agriculture more comprehensively by researching these five provinces.

Secondly, the five provinces have a large grain crop output, accounting for 20.6% of China’s total grain output in 2019, and the agriculture is relatively developed. Thirdly, the five provinces have an advanced agricultural waste recycling system. Taking straw utilization as an example, its overall comprehensive utilization rate is as high as 80%. Besides this, the demonstration work of agricultural ecological protection is excellent as well [37], which provides a certain investigation basis and research value. Some scholars have also focused on the performance of agricultural waste utilization in the five provinces with topics of discussion such as those on waste incineration and disposal [38], and analyses on waste biological disposal strategy [39]. However, there are still some problems, such as the single research object (taking a single Province as the research object), and a focused research perspective (focusing on the topic of utilization mode), etc. What this means is that green utilization of agricultural waste has not been studied from the perspective of farmers.

#### 3.1.2. Data Collection and Statistics on Respondents

Generally, the sample size of the scale type questionnaire is about 20 times the scale items. This study involves 41 items, so the sample size should be larger than 820. Since the research subjects are farmers and factors such as questionnaire quality and willingness to participate must be considered, the sample size is 1000 to avoid the decline of sample quality caused by too many invalid questionnaires. In the sample selection, since the number of farmers in the five provinces is not too different, we determined 200 samples for each province. Secondly, through discussion with government staff, we selected the representative cities of agriculture in each province for the questionnaire: Nantong, Yancheng, and Huai’an in Jiangsu; Wuhu, Hefei, and Anqing in Anhui; Xi’an, Baoji, and Xianyang in Shaanxi; Lanzhou, Baiyin, and Dingxi in Gansu; Chengdu, Mianyang, and Suining in Sichuan. Finally, a cluster sampling method was adopted to distribute questionnaires to representative villages and towns.

This study took the green utilization of straw as the research event. A structured questionnaire —as the primary research instrument—was given to 1000 participants in the five provinces from March to July 2019. The questionnaires consisted of 41 items with responses recorded on a 5-point Likert scale, with options ranging from “completely disapproved” (score = 1) to “fully approved” (score = 5). The response rate was 82.7% (827 questionnaires were taken back). After eliminating invalid questionnaires, 704 valid questionnaires were obtained, and the effective rate was 85.1%.

The subjects of the survey are those who live in rural areas and make their living mainly through agricultural production activities. In the questionnaire design, gender, age, educational background, family structure, and other indicators are also considered. The basic information of the sample is as follows: 65.82% of interviewees were male farmers, around 49 years old on average. Furthermore, 78.67% of the farmers had a junior high school degree or below. Besides this, 43.2% of the farmers go out as migrant workers, which puts more pressure on agricultural labor. In terms of the household structure, the percentage of families with 3–5 persons’ accounts for 73.2% of the sample, and the average agricultural income is about 17,300 yuan.

### 3.2. Descriptive Analysis

From the perspective of farmers’ “motivation-cognition-behavior,” this paper verifies the influence mechanism of green utilization of agricultural waste. Considering that various motivational factors as well as cognitive and behavioral sub-dimensions are all typical latent variables that are difficult to observe and measure directly, the paper introduces measurable variables to conduct actual measurements [40]. Among them, the determination of the measurable variables of a positive and negative broken window is obtained by the author’s analysis of the phenomenon of the broken window effect and through interviews with experts. The determination of measurable variables in other sub-dimensions rely on document [24], document [30], and document [41] for reference. The descriptions of latent variables, measurable variables, and samples are shown in Table 1.

All indicators will also be affected by the age of farmers. We divided the age of the respondents into six groups: 20–29; 30–39; 40–49; 50–59; 60–69; and 70 and above, and analyzed the standard deviation of the measured variables of each age group in each test item. We found the following: (1) the top five dimensions that are most affected by age were Return (0.533) (the mean standard deviation in brackets), Positive Broken Window Effect (0.523), Negative Broken Window Effect (0.448), Response Cost (0.427), and Subjective Norms (0.363); and (2) that young people (20–29 years old, 30–39 years old) can quickly participate in the green disposal of waste—their practical abilities, such as learning, are more prominent, and their resistance to bad behaviors is also greater. The older farmers (50–59 and 60–69 years old) are more likely to get external support and form better subjective norms, but they are also more likely to be affected by external motivation and produce more negative emotions. The elderly farmers (over 70 years old) are more vulnerable to the impact of policies and systems. Due to their age, their practical and learning abilities are insufficient.

## 4. Examination of Influencing Factors of Green Utilization of Agricultural Waste

Considering the multiple latent variables to be dealt with and the implicit relations among each latent variable and certain errors in the measurements of farmers’ motivation, cognition, and behavior, a structural equation model is used in this paper. In order to eliminate the interference among multiple variables, we allowed measurement errors to exist between independent and dependent variables to make the research results more realistic and practical, and to obtain the influence paths of various factors for green utilization of agricultural waste [42,43]. The path relations among thirteen types of latent variables are discussed in order to obtain the influence mechanism of green utilization of agricultural waste. AMOS21 (IBM, Armonk, NY, USA) is the suitable software to deal with the structural equation model, so we used it to test the influencing factors.

### 4.1. Reliability and Validity Test

A correlation test of 41 items of the questionnaire was carried out. The Cronbach’s Alpha values of all items are higher than 0.6, and the Cronbach’s Alpha values of 13 latent variables are all above 0.7. The consistency and stability of the questionnaire are good, and the items are convincing. In the KMO and Bartlett’s test, the KMO coefficient of the sample reaches 0.78. It is significant at the level of 0.01, indicating that the overall structure of the variable is reasonable, and the reliability is high, which is suitable for structural equation analysis. Through Exploratory Factor Analysis (EFA), this paper tests the common method bias (CMB) of the questionnaire based on Harman’s single-factor method [44], in which the single factor interpretation amount is 28.513%, less than 40% of the judgment limit, so the CMB problem is not significant, which can be further studied.

### 4.2. Result Analysis

Based on AMOS21, the goodness-of-fit index is carried out on the model. The results show that the RMESA and RMR are 0.043 and 0.026, respectively, which are closer to the ideal state of 0. Goodness-of-Fit-Index(GFI), Comparative-Fit-Index(CFI), and Normed-Fit-Index(NFI) are 0.936, 0.944, and 0.917, respectively, and are also at ideal levels, indicating that the fitting degree between the model and the sample is high, and the overall reliability is strong. In the structural equation analysis, in order to ensure the stability of the parameter estimation value, it is necessary to carry out standardized processing, and finally, to get the model calculation results such as the standard weight coefficient. The results are shown in Table 2.

**Figure 2:**
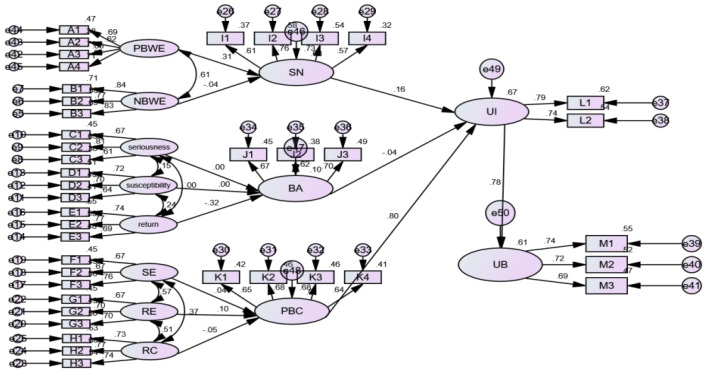
Structural model of the green utilization factors of agricultural waste. Notes: PBWE: positive broken window effect; NBWE: negative broken window effect; SE: self-efficacy; RE: response efficacy; RC: response cost; SN: subjective norms; BA: behavioral attitude; PBC: perceived behavioral control; UI: utilization intention; UB: utilization behavior.

Combining the analysis framework of Figure 1 and the standardized coefficient of the effect pathway in Table 2, we finally obtain the influence relations of the variables related to green utilization of agricultural waste, as shown in Figure 2. The “motivation-cognition-behavior” structural model reflects how that farmers’ straw utilization is influenced by intrinsic and extrinsic motivations and protection motivations.

The hypothetical results are shown in Table 3:

The path relations in the “motivation-cognition-behavior” structural model are as follows:

(1) In the path of “extrinsic motivation → subjective norms.” The positive broken window effect and negative broken window effect have significant positive and negative correlations with farmers’ subjective norms, respectively, and their standardized regression coefficients are 0.314 and −0.047, respectively. It is shown that the broken window effect plays an important role in promoting the creation of a good external environment for the green utilization of agricultural waste. In contrast, the negative broken window effect does hinder the creation of a green utilization atmosphere. However, the negative effect is relatively weak, which is basically in accord with the current situation in rural areas. From the perspective of farmers’ perception of the broken window effect, farmers’ acceptance of a positive broken window effect is higher than perceptions of a negative one. Farmers are more inclined to “positive activities that can generate benefits,” that is, they attach great importance to the benefits obtained from green utilization activities. Therefore, strengthening the positive broken window effect and establishing positive models are also important regulatory measures to eliminate the spread of bad behaviors.

(2) In the path of “intrinsic motivation → behavioral attitude.” The seriousness has a significantly positive correlation with the behavioral attitude of farmers, and its standardized regression coefficient is 0.004; the susceptibility and return have significantly negative correlations with behavioral attitude, and its standardized regression coefficient is 0.004 and −0.314, respectively. It is shown that the higher the degree of farmers’ awareness of the seriousness of the negative disposal of agricultural waste, the more likely it is that farmers will be inclined to green utilization of agricultural waste, but the relation coefficient between the two is small. It is likely that under the premise of the existence of negative broken window activities, some farmers are influenced by the “negative broken window effect.” As a result, they do not form the inherent motivation of green utilization but choose to use the loopholes in environmental governance to obtain benefits. On the contrary, the more return farmers got from the negative disposal of agricultural waste, the weaker the intrinsic motivation for participating in green utilization was. Moreover, the susceptibility of farmers to bad behaviors could increase their motivation for participation. However, farmers’ sensitivity to bad behaviors is abated by the impact of the negative broken window effect, which contributes to a weak negative impact of susceptibility on farmers’ behavioral attitudes.

(3) In the path of “response analysis → perceived behavioral control.” The self-efficacy and response efficacy have significantly positive correlations with the behavioral attitude of farmers, and their path coefficients are 0.048 and 0.129, respectively. It shows that the more thoroughly farmers analyze their capabilities, and the more they understand the positive meaning of green utilization of agricultural waste, the higher the degree of farmers’ controlling green utilization behaviors. At the same time, due to the potential contribution of the positive broken window effect, the effect of response efficiency on farmers is higher than that of self-efficacy. However, the response cost harms the perceived behavioral control, with the path coefficient being −0.053. The higher the cost of utilization, the more likely farmers will find it challenging to have the ability and conditions to participate in the green utilization of agricultural waste.

(4) In relation to the “cognitive dimension → behavioral dimension.” The subjective norms of farmers have a significantly positive impact on utilization intention, and the path coefficient is 0.164. This shows that the positive broken window effect dominates the farmers’ psychology, and can promote the green utilization of agricultural waste by cultivating positive extrinsic motivation and optimizing the green utilization environment. The behavioral attitude has a negative impact on utilization intention, with the path coefficient being −0.038, which indicates that the behavioral attitude of farmers at this stage is not conducive to the cultivation of utilization intention. This mainly results from farmers’ poor awareness of the seriousness of the negative disposal of agricultural waste. Besides, due to the interference of negative broken window activities, farmers can be more sensitive to susceptibility and return. Perceived behavioral control plays an important role in promoting the utilization intention, with the standardized regression coefficient reaching 0.595. It shows that farmers have fully understood their abilities and can stably control their utilization activities. The utilization intention has a positive correlation with utilization behavior, with the standardized regression coefficient reaching 0.583. It indicates that the utilization intention dominates the utilization behavior, that is to say, for farmers, there is no deviation between their intention and behavior.

(5) In the relation path of “measurable variables → latent variables.” Benefits-oriented publicizing (A3) has the most impact on the “positive broken window,” with a path coefficient of 0.845. The path coefficients of positive examples (A1), no incineration supervision (A2), and active publicity (A4) are 0.686, 0.617, and 0.842, respectively. Therefore, in the process of green utilization of agricultural waste, farmers’ benefits should be guaranteed by adopting effective recycling methods and providing effective economic incentives, etc. By doing so, we can establish a positive broken window mechanism to cultivate farmers’ subjective norms and utilization intention. The supervision loopholes (B1) impose the most significant impact on the “negative broken window,” with a path coefficient of 0.843, indicating that regulation loopholes and poor supervision can easily cause the spread of a “negative broken window” effect. In terms of seriousness, the seriousness of health (C2) is its important influencing factor, and the path coefficient is 0.807.

It shows that farmers are more concerned about the threat of bad behavior to their health, indicating that farmers have recognized the relationship between negative disposal of agricultural waste and health. From the perspective of susceptibility, the impact of image loss (D1) is quite huge (path coefficient is 0.716), showing that the negative disposal of agricultural waste is the leading cause of the deteriorated image of villages and towns, which in turn affects the economy and ecological maintenance. From the perspective of return, time return (E2) is an important influencing factor, and the path coefficient is 0.77. At the same time, money return (E1) (path coefficient is 0.739) also induces farmers to adopt bad disposal behaviors, which reflects that time and money costs are the main factors preventing farmers from participating in the green utilization of agricultural waste. Correspondingly, time efficacy (F3) has the strongest impact on farmers’ self-efficacy (path coefficient is 0.761), and time cost (H2) has the most significant impact on farmers’ response cost (path coefficient is 0.775), and self-response (G3) produces a strong response efficacy (path coefficient is 0.705).

It demonstrates that in the process of green utilization of agricultural waste, farmers are eager to get health and soil returns. However, the limited economic strength and their worries about the lack of time or the high cost of time make it difficult for farmers to conduct green utilization activities. Family support (I2) and rural neighbor support (I3) have the most significant impact on the formation of farmers’ subjective norms (path coefficients are 0.759 and 0.733, respectively). They influence the attitudes of family members and rural neighbors through positive broken window activities, which further affect their extrinsic motivations, thus enhancing subjective norms. Recognizing the fact that agricultural waste can be beneficial to the resource (J3) helps to improve farmers’ behavioral attitude (path coefficient is 0.701), while focusing on an improvement of learning ability (K2) and cognition of policy (K3) (path coefficients are 0.677 and 0.678, respectively) directly leads to the enhancement of farmers’ perceived behavioral control.

## 5. Conclusions

Based on motivation theory and the theory of planned behavior, this paper has explored the influence mechanism of green utilization of agricultural waste from the perspective of farmers. Main works are as follows:

Establishing the “motivation-cognition-behavior” analysis framework.

Analysis of the motivational dimension from the perspective of extrinsic motivation, intrinsic motivation, and response motivation that can better clarify the impact of farmers’ psychological decisions on the green behavior of agricultural waste. In the cognitive dimension, three sub-dimensions of subjective norms, behavioral attitude and perceived behavioral control are considered. They are influenced and controlled by multiple motivations. The utilization intention and utilization behavior are discussed in the behavioral dimension to represent the actual behavior of farmers.

Defining various variables in the “motivation-cognition-behavior” analysis framework of farmers (i.e., eight motivational factors, three cognitive sub-dimensional variables, and two behavioral sub-dimensional variables).

In the extrinsic motivation dimension, the broken window effect is introduced to explain the farmers’ behavior because the particularity of the farmer group determines that the external interference will have a significant influence on their psychological decision-making. It is believed that in the green utilization of agricultural waste, there is a negative broken window effect and a positive broken window effect existing in the farmer group, that is, the negative transmission mechanism and the positive transmission mechanism.

The AMOS21 was used to analyze the structural equation model and measure the relations between various factors.

The study found that: (1) In the “motivation dimension → cognitive dimension” relation, the broken window effect of extrinsic motivation has a significant effect on the process of formation of farmers’ subjective norms. The negative broken window effect has a significant negative influence, while the positive broken window effect has a positive influence, which has a more significant impact on farmers’ attitudes. In the intrinsic motivation, farmers form negative behavioral attitudes through a threat analysis of negative disposal of agricultural waste. The return dominates the formation of farmers’ behavioral attitudes. The seriousness promotes the changes of behavioral attitudes, but the effect is weak. Susceptibility affected by the impact of the broken window effect has a negative effect on farmers’ participation attitudes.

It indicates that farmers’ attitudes towards the utilization of agricultural waste is not determined at this stage, and the vast benefits and convenience of waste incineration are still interfering with farmers’ behavior. Therefore, the regulation loopholes will trigger negative broken window activities and induce farmers to adopt negative disposal behavior. In the response analysis, farmers can know their capacity status. Self-efficacy and response efficacy dominate the farmers’ response motivation of waste utilization, and a positive consensus is achieved, which gives farmers the confidence and ability to conduct green utilization of agricultural waste. However, due to the high response cost of the green utilization of agricultural waste, the farmers’ participating desire is restrained to a certain extent, and some farmers are waiting to see what will happen next. (2) In the “Cognitive dimension → behavior dimension” relation, except for behavioral attitudes, which negatively interfere with the utilization intention, subjective norms and perceived behavioral control all contribute to the promotion of utilization intention, which then effectively promotes the utilization behavior.

This study is based on the behavior of farmers in five provinces of China, which have certain representativeness. First, Jiangsu and Anhui reflect the agricultural situation of the plain, Shaanxi and Gansu reflect the agricultural characteristics of the central plateau, Sichuan reflects the agricultural situation of the basin, and the five provinces cover the main planting terrain and crop types in China. Second, the grain output of the five provinces is over 20% of that of the whole country, which is an important agricultural area. Third, five provinces pay more attention to the green utilization of agricultural waste, which covers the main utilization mode of agricultural waste in China. Through the study of the above five provinces, we can more truly reflect the overall situation of green utilization of agricultural waste in China. For developing countries, the research conclusions also have some reference value. Based on the verification results of farmers’ “motivation-cognition-behavior” structural model, this paper believes that policy optimization should focus on farmers’ motivations in order to enhance farmers’ subjective norms, behavioral attitudes, and perceived behavioral control. The specific suggestions are as follows:

(1) Based on the influence of farmers’ extrinsic motivation on subjective norms, attention should be paid to enhancing the positive broken window effect and reducing the negative one. On the one hand, farmers’ behavior is affected by government leadership and farmer groups, and the influence of farmer groups is significantly greater than policy regulations and incentives. Therefore, we must pay attention to building an information exchange platform for farmer groups, and enable government personnel and enterprise personnel to closely connect with farmers in order to regularly explain the relevant knowledge about green utilization of agricultural waste, eliminate the negative thoughts of “incineration causes no damage,” and supervise farmers’ behavior. On the other hand, we must improve the incentive system and cultivation system for the green utilization of agricultural waste, with special attention paid to the protection of farmers’ interests.

Moreover, we must encourage and help farmers who are eager to participate in green utilization activities, including financial support, spiritual guidance, policy support, and enterprise participation, etc., to eliminate the inadaptability of farmers’ initial participation, and give rewards to farmers who actively publicize green utilization and resist incineration. Furthermore, we must improve the speaking power of these farmers by setting up public service posts, or promote the publicity of opinion leaders, thereby strengthening the effect of the positive broken window to guide farmers towards turning their participating intention into practical action. Additionally, we must establish a comment and opinion wall for the green utilization of agricultural waste to improve the recognition and reputation of green utilization behavior, thus attracting more farmers to take the initiative to participate. At the same time, we must reduce the effect of a negative broken window in policy-making, and strengthen the construction of the supervision system for agricultural waste incineration. We can guide those farmers who burn waste for the first time or those who do not know the policy with publicity and education, and severely punish those who repeatedly adopt incineration and persuade others to do so, thus effectively curbing the negative effect of the negative broken window effect on farmers’ extrinsic motivation.

(2) Based on the influence of farmers’ intrinsic motivation on behavioral attitudes, attention should be paid to enhancing farmers’ awareness of the seriousness of incineration behaviors, optimizing farmers’ susceptibility to incineration behaviors, and suppressing its high return. Due to the information asymmetry, it is difficult for farmers to understand the great damage caused by incineration activities. Therefore, the government must publicize the losses of agricultural waste incineration over the years, focus on publicizing the negative impact of incineration on farmers’ health, and popularize related penalty policy to help farmers re-examine the seriousness of waste incineration. In order to strengthen farmers’ perception of incineration behavior, the government can first establish files of points recording incineration and utilization times, thus linking family image and village image, personal interests, and environmental interests. Second, let farmers understand the differences between utilization and incineration and recognize the direct damage caused by incineration. Lastly, we can enhance farmers’ perception that waste utilization can improve rural image, income, and health. In the cognition → behavior path, only behavioral attitudes have a weak negative effect on the utilization intention, which demonstrates that farmers’ behavioral attitude is still negative in the short term, so stimulating farmers’ intrinsic motivation is an important task at present.

(3) Based on the impact of farmers’ response motivation on perceived behavioral control, attention should be paid to enhancing farmers’ self-efficacy and response efficacy in the green utilization of agricultural waste and on a reduction of response costs. Effective participation capabilities have been formed, but farmers still worry too much about high participation costs. Therefore, it is necessary to improve the infrastructure of green utilization of agricultural waste, optimize inefficient loopholes, support the scale, mechanize an intelligent development of agricultural waste recycling, and alleviate the pressure of high time costs and shortage of hands. At the same time, we can help farmers understand green utilization technology, highly publicize the sustainable effects of green utilization, and guide farmers to view green utilization more comprehensively and scientifically. By doing so, we can enable a change in farmer attitudes from “want to participate” to “be able to participate,” and lay a solid foundation for the mass base of a utilization and participation group.

(4) Last but not least, as this study found, age is a factor affecting farmers’ behavior. It is thus suggested that different age groups of farmers’ behavior should be considered in policymaking and implementation. For example, it is important to guide young people with a strong learning ability to accept the green use of agricultural waste and promote young people to take the lead in the green use of agricultural waste by means of training, guidance, and equipment support, and in other ways, to widely publicize the successful experience of young people in order to promote the participation of farmers from other age groups. Secondly, it is necessary to have a special dialogue with seniors or family farm leaders and to educate them, encourage them to try to practice a green use of agricultural waste, and to guide the whole family to participate with their leaders. Finally, the economic incentive policy should focus on farmers between 50 and 69 years of age. Its implementation reduces the concerns about the cost of participation, hence enhancing the willingness to participate.

## Figures and Tables

**Table 1:** Definition and Descriptive Statistics.

Latent Variables	Measurable Variables		Measurement Content	Average	Standard Deviation
**Positive Broken Window Effect**	A1	Positive Examples	If someone participates in the green disposal of straw and gains a lot, I will envy or also want to participate	3.58	0.936
	A2	No Incineration Supervision	If no one incinerates or discards the straw, I will also refuse to do it or dare not incinerate	3.82	0.873
	A3	Benefits-Oriented Publicity	If I gain benefits in the green utilization of straw, I will also publicize the benefits and persuade others to do so	3.41	0.891
	A4	Active Publicity	If I have explicitly refused to incinerate straw, then I will also supervise others or lead them to resist straw incineration	3.11	0.957
**Negative Broken Window Effect**	B1	Supervision Loopholes	If others incinerate the straw without punishment, I may also do it	2.76	0.849
	B2	Vacant System	I may incinerate straw if there is no corresponding punishment or supervision mechanism	3.04	0.948
	B3	Publicizing for Incineration	If I incinerate straw without being punished, I might also persuade others to do so	2.46	0.92
**Seriousness**	C1	Seriousness of Environment	It is believed that straw incineration will affect the environment, economy and village image	3.52	0.871
	C2	Seriousness of Health	It is believed that straw incineration will affect one’s health	3.75	0.761
	C3	Seriousness of Punishment	It is believed that incinerating straw will be punished	3.89	0.758
**Susceptibility**	D1	Image Loss	Possibility of straw incineration affecting environment, economy and village image	3.37	0.802
	D2	Loss of Health	Possibility of straw incineration affecting health	3.58	0.745
	D3	Money Loss	Possibility of punishment	3.81	0.758
**Return**	E1	Money Return	Incinerating straw costs less money	2.85	1.005
	E2	Time Return	Incinerating straw costs less time	3.05	0.951
	E3	Energy Return	Incinerating straw costs less energy	3.16	0.94
**Self-Efficacy**	F1	Capability Efficacy	Having the ability to conduct green utilization of straw	3.59	0.86
	F2	Money Efficacy	Having money to conduct green utilization of straw	3.52	0.841
	F3	Time Efficacy	Having time to conduct green utilization of straw	3.36	0.919
**Response Efficacy**	G1	Environment Response	Green disposal of straw can optimize the environment and promote economic development	3.47	0.878
	G2	Agriculture Response	Green disposal of straw can promote sustainable development of agriculture	3.57	0.822
	G3	Self-Response	Green disposal of straw can protect one’s health and prevent soil pollution	3.6	0.834
**Response Cost**	H1	Money Cost	Green disposal of straw costs more money	2.92	0.879
	H2	Time Cost	Green disposal of straw costs more time	3.07	0.855
	H3	Energy Cost	Green disposal of straw costs more energy	3.17	0.864
**Subjective Norms**	I1	Leader Support	Support and recognition from village leaders	3.76	0.783
	I2	Family Support	Support and recognition from family	3.45	0.925
	I3	Neighbor Support	Support and recognition from neighbors	3.45	0.944
	I4	Society Support	Green disposal of straw meets social trends and national requirements	3.74	0.826
**Behavioral Attitude**	J1	Economically Beneficial	It is believed that green disposal of straw can increase household income	3.74	0.795
	J2	Environmentally Beneficial	It is believed that green disposal of straw can promote sustainable ecological development and the transformation of green agriculture	3.38	0.86
	J3	Beneficial to Resource	It is believed that the green disposal of straw can solve the problem of idle straw and make full use of production resources	3.68	0.864
**Perceived Behavioral Control**	K1	Financial Ability	I have the financial ability to invest the time and energy	2.94	0.943
	K2	Leaning Ability	I have the ability of independent learning	3.01	1.001
	K3	Cognition of Policy	I am familiar with the policies and channels	3.49	0.863
	K4	Independence	I can independently decide how to dispose of the straw in a green way	3.67	0.809
**Utilization Intention**	L1	Utilization Intention	Whether you are willing to participate in green disposal of straw or not	3.04	0.82
	L2	Utilization Trend	Whether you are willing to learn related knowledge and policies or hold a positive attitude toward green utilization	3.51	0.786
**Utilization Behavior**	M1	Behavior State	Whether the green disposal has started	3.39	0.752
	M2	Channel State	Whether a channel of green disposal has been opened or the corresponding equipment has been purchased	2.98	0.985
	M3	Technical State	Whether you have learned or mastered relevant techniques, policies and so on	3.33	0.91

**Table 2:** Estimated Results of Model Path Coefficient.

Effect Pathway	Standardized Regression Coefficient	Standard Error	T-Value	Significance Level
		S.E.	C.R. (Critical ratio)	*p*
Subjective norms<---positive broken window effect	0.314	0.048	4.845	***
Subjective Norms<---negative broken window effect	−0.047	0.041	−3.766	***
Behavioral Attitude<---seriousness	0.004	0.046	4.018	***
Behavioral Attitude<---susceptibility	−0.004	0.05	−3.874	***
Behavioral Attitude<---return	−0.314	0.04	−5.629	***
Perceived Behavioral Control<---self-efficacy	0.048	0.079	1.715	**
Perceived Behavioral Control<---response efficacy	0.129	0.087	4.294	***
Perceived Behavioral Control<---response cost	−0.053	0.063	−1.545	**
Utilization Intention<---subjective norms	0.164	0.053	4.075	***
Utilization Intention<---behavioral attitude	−0.038	0.048	−1.895	**
Utilization Intention<---perceived behavioral control	0.595	0.063	11.523	***
Utilization Behavior<---utilization intention	0.583	0.046	11.621	***
A1<---positive broken window effect	0.686	0.046	14.623	***
A2<---positive broken window effect	0.617	0.057	14.707	***
A3<---positive broken window effect	0.845	0.061	19.168	***
A4<---positive broken window effect	0.842	0.066	19.127	***
B1<---negative broken window effect	0.843	0.063	21.972	***
B2<---negative broken window effect	0.768	0.047	21.567	***
B3<---negative broken window effect	0.829	0.046	23.119	***
C1<---seriousness	0.67	0.072	12.273	***
C2<---seriousness	0.807	0.085	12.369	***
C3<---seriousness	0.613	0.063	12.616	***
D1<---susceptibility	0.716	0.071	12.539	***
D2<---susceptibility	0.702	0.073	12.408	***
D3<---susceptibility	0.641	0.069	12.271	***
E1<---return	0.739	0.068	14.992	***
E2<---return	0.77	0.064	15.35	***
E3<---return	0.691	0.058	14.951	***
F1<---self-efficacy	0.669	0.076	13.323	***
F2<---self-efficacy	0.672	0.073	13.503	***
F3<---self-efficacy	0.761	0.087	14.011	***
G1<---response efficacy	0.673	0.069	13,794	***
G2<---response efficacy	0.703	0.071	13.824	***
G3<---response efficacy	0.705	0.072	13.837	***
H1<---response cost	0.731	0.064	16.015	***
H2<---response cost	0.775	0.063	16.478	***
H3<---response cost	0.736	0.061	16.183	***
I1<---subjective norms	0.608	0.111	12.648	***
I2<---subjective norms	0.759	0.109	13.583	***
I3<---subjective norms	0.733	0.108	13.457	***
I4<---subjective norms	0.565	0.085	11.495	***
J1<---behavioral attitude	0.67	0.091	11.303	***
J2<---behavioral attitude	0.62	0.089	11.279	***
J3<---behavioral attitude	0.701	0.1	11.345	***
K1<---perceived behavioral control	0.645	0.078	13.927	***
K2<---perceived behavioral control	0.677	0.079	14.033	***
K3<---perceived behavioral control	0.678	0.069	14.036	***
K4<---perceived behavioral control	0.645	0.063	13.556	***
L1<---utilization intention	0.789	0.07	17.845	***
L2<---utilization intention	0.736	0.05	17.82	***
M1<---utilization behavior	0.743	0.082	16.739	***
M2<---utilization behavior	0.718	0.078	16.137	***
M3<---utilization behavior	0.688	0.072	15.632	***

Note: ***, **, indicate significant level at 1%, 5% respectively.

**Table 3:** Hypothetical results.

Hypothesis	Assumption Content	Result
H1	External motivation has a positive effect on farmers’ subjective norms	
H1a	Positive broken window has a positive effect on subjective norms	Accept
H1b	Negative broken window has a negative effect on subjective norms	Accept
H2	Intrinsic motivation (threat assessment) has a positive effect on the behavior attitude of farmers	
H2a	Seriousness has a positive effect on behavior attitude	Accept
H2b	Susceptibility has a positive effect on behavior attitude	Refuse
H2c	Return has a negative effect on behavior attitude	Accept
H3	Response analysis has a positive effect on the perceived behavior control of farmers	
H3a	Self-efficacy has a positive effect on perceived behavior control	Accept
H3b	Response efficacy has a positive effect on perceived behavior control	Accept
H3c	Response cost has a negative effect on perceived behavior control	Accept
H4	Cognition has a positive effect on behavior	
H4a	Subjective norms have a positive effect on utilization intention	Accept
H4b	Behavior attitude has a positive effect on utilization intention	Refuse
H4c	Perceived behavior control has a positive effect on utilization intention	Accept
